# Quality of Life of Patients After an Acute Coronary Event: Hospital Discharge

**DOI:** 10.14740/jocmr1865w

**Published:** 2014-07-28

**Authors:** Cristiane Maria Carvalho Costa Dias, Luciana Bilitario Macedo, Lilian Tapioca Jones Cunha Gomes, Paula Luzia Seixas Pereira de Oliveira, Iana Verena Santana Albuquerque, Amanda Queiroz Lemos, Cristina Aires Brasil, Eloisa Pires Ferreira Prado, Pedro Santiago Macedo, Francisco Tiago Oliveira de Oliveira, Helena Franca Correia dos Reis, Eduardo Sahade Darze, Armenio Costa Guimaraes

**Affiliations:** a Bahiana School of Medicine, Salvador, Bahia, Brazil; b Hospital Alianca, Salvador, Bahia, Brazil; c ASSOBRAFIR, Salvador, Bahia, Brazil; d State University of Bahia, Salvador, Bahia, Brazil; e Hospital Cardio Pulmonar, Salvador, Bahia, Brazil; f Hospital Santa Izabel, Salvador, Bahia, Brazil; g Federal University of Bahia, Brazil; h Hospital Geral do Estado, Brazil

**Keywords:** Life quality, Acute coronary syndrome, Cardiovascular rehabilitation 1

## Abstract

**Background:**

The acute coronary syndrome (ACS) has a high morbi-mortality rate, including physical deficiencies and functional limitations with impact on quality of life. Cardiovascular rehabilitation 1 (CVR1) should begin as early as possible, to enable improvement in functional capacity and quality of life. Previous studies have shown association of cardiovascular diseases with quality of life, in which depression and anxiety are the domains most altered. The aim of the study is to verify the impact of an acute coronary event on quality of life at the moment of hospital discharge.

**Methodology:**

This was a cross-sectional study, with ACS patients hospitalized in ICU of a private hospital in the city of Salvador, Brazil, submitted to CVR1. The quality of life questionnaire Euroqol-5D was applied on discharge from hospital. Patients included in the study were those with ACV, who had medical permission to walk, had not been submitted to acute surgical treatment, were time and space oriented, and over the age of 18 years. Patients excluded from the study were those with cognitive, orthopedic and neurological problems, who used orthesis on a lower limb, and were in any condition of risk at the time of beginning with CVR1. Data were collected by a previously trained ICU team.

**Results:**

Data were collected of 63 patients who revealed compromise in the domains of pain/feeling ill (20.63%) and anxiety/depression (38.09%). Statistical significance was observed in the association between sex and pain/feeling ill (P < 0.01), sex and anxiety/depression (P < 0.01), diabetes and mobility (P < 0.01), hereditary factors and anxiety/depression (p < 0.01), BMI and pain/feeling ill (P < 0.01).

**Conclusion:**

In this sample of patients, on discharge from hospital after ACS, the pain/feeling ill and anxiety/depression domains were shown to be compromised.

## Introduction

The acute coronary syndrome (ACS) is a disease occurring with high frequency, associated with a high rate of morbi-mortality, causing physical deficiencies and functional limitations with impact on the quality of life (QoL) [1]. According to data from the Informatics Department of the Brazilian National Health System “Sistema Unico de Saude” (DATASUS), in the year 2010 the majority of deaths were caused by circulatory system diseases. A high number of deaths in the country (99,995) were due to ischemic heart disease. Irrespective of the socioeconomic class and region of Brazil, this is the cause of the highest number of deaths in the population [2].

ACS is caused by coronal obstruction of the interaction between the phenomena of thrombosis and vasospasm. It comprises a group of entities that include acute myocardial infarction (AMI) with ST segment elevation (STEMI), AMI without ST segment elevation (NSTEMI) and unstable angina (UA) [3, 4]. The therapeutic treatment after the acute coronary event comprises medication treatment, highly complex technology (angioplasty or surgical treatment) and cardiovascular rehabilitation 1 (RCV1) [5]. This arsenal of treatment comprises the period from the time of onset of the coronary event through to the patient’s discharge from hospital, and must be instituted as early as possible. The rehabilitation program enables an improvement in functional capacity and in the QoL of this population [6, 7].

According to the World Health Organization (WHO), since 1995, QoL is translated as “the individual’s perception of his/her position in life, within the context of culture and value systems in which he/she lives and in relation to his/her objectives, expectations standards an concerns” [8]. However, this understanding has been discussed, both changing and amplifying its concept, which includes a range of conditions that may affect the individual’s perception, feelings and behaviors related to his/her daily activities, health conditions and medical interventions [9]. By evaluating QoL, one has knowledge of the functional status, impact, limitation treatment conditions and perspective of life of individuals [8].

At present, a close relationship between cardiovascular diseases and QoL has been observed, in which these diseases may have an impact on QoL. Studies have proved that depression is generally present before the coronary event, thus being considered as another risk factor for the pathology. The domain of anxiety has been shown to be present after the coronal event, since this disease represents the threat of death [10, 11].

Bearing in mind that ACS impacts on the QoL of these patients, generating biopsychosocial changes, this study is relevant, because there is a lack of researches that investigate the QoL of patients with ACS on discharge from hospital. This investigation may contribute towards better knowledge of the factors that influence the QoL of this population, and it is our hope that we will be able to base innovative protocols in RCV1 on this research, and consequently reduce this impact. Thus, the aim of this study was to verify the impact of an acute coronary event on QoL at the moment of hospital discharge.

## Methodology

This was a cross-sectional study in patients with ACS submitted to a progressive exercise program in CVR1, hospitalized in the ICU in a private hospital in the city of Salvador, Brazil, in the period of October 2011 to December 2012. Data collection was performed by physical therapists in the ICU of the hospital where the research was conducted. Patients included in the study were those with ACV, who had medical permission to walk, had not been submitted to acute surgical treatment, were time and space oriented, and over the age of 18 years. Patients excluded from the study were those with cognitive, orthopedic and neurological problems, who used orthesis on a lower limb, and were in any condition of risk at the time of applying the TC 50m, which in the opinion of the physical therapist, might place the patient in a situation of risk [12]. The sample calculation was performed using the “LEE” calculator of the Epidemiology and Statistics Laboratory at the University of Sao Paulo (USP) (“Laboratorio de Epidemiologia e Estatistica”, USP). It was based on a previous study in which the impact of chronic heart failure on the QoL of patients was verified, and on the comparison of two proportions: proportion group 1 (69.9%) and proportion group 2 (46.2%), with 71 patients being required, with an alpha type error of 5% and test power of 80%. Considering a loss of 20%, the final sample would be 57 patients [13].

The stages of the research were composed of the application of the sociodemographic questionnaire, a clinical evaluation, performance of RCV1, in which the patients would perform progressive low intensity exercises in accordance with the individual cardio-circulatory responses, and afterwards, the application of the instrument Euroqol-5D. This instrument was used to evaluate the QoL of these patients, bearing in mind that it was validated in a previous study because it was an instrument that was easy to apply and sensitive for this population [1]. It is composed of five questions that cover the following domains of health: mobility, personal care, habitual activities, pain/feeling ill and anxiety/depression, classified into the following three categories: without problems, moderate problems and extreme problems. The score awarded to the responses varies according to the alternatives and domains. Thus, if an individual has a score of 100% in the evaluation, this would be considered an excellent QoL; that is, without problems. This instrument was given to the patient, who was instructed to answer it at the time of being discharged from hospital.

The following were the independent variables of the study: sex, body mass index (BMI) in accordance with the WHO [14], ACS classification, type of treatment, dyslipidemia, hypertension, hereditary factor for early death, diabetes and smoking. The dependent variables in the Euroqol-5D domains were: mobility, personal care, habitual activities, pain/feeling ill and anxiety/depression.

The descriptive and analytical analysis was performed by means of the database, using the Statistical Package for Social Sciences software program (SPSS), version 14.0 for Windows. The analysis of normality of the variables was performed by the Kolmogorov-Smirnov test. The results were presented in Tables and/or Figures, the categorical variables were expressed as frequency (%). The analysis of continuous variables with normal distribution was express as mean and standard deviation (X ± SD). The test used for comparison of the categorical variables was the Chi-square, and when this was not suitable, the exact Fischer test was used. The level of significance adopted was P ≤ 0.05.

This study was submitted to the Research Ethics Committee of EBMSP Protocol No. 170/2011. The patients admitted to the ICU were provided with explanations about the objectives of the research, and if they decided not to participate, their decision was respected. At any time, the patient could have access to the professionals responsible for the research for explanations of any doubts that might arise. At the time of discharge, each patient was aware of the results and received guidance about the need to continue practicing progressive exercises in the extra-hospital stage of rehabilitation.

## Results

Sixty-three patients were evaluated. Of these, 25 (39.7%) suffered acute myocardial infarction without STEMI (NSTEMI), 15 (23.8%) AMI with STEMI and 22 (34.9%) with unstable angina (UA). The male sex predominated, 44 (69.8%), with a mean age of 59.16 ± 12.15 years. As regards treatment, 37 (64.9%) received clinical treatment and 20 (35.0%) were submitted to angioplasty. The mean weight of patients was 77.52 kg (± 15.63), mean height 1.69 m (± 0.09) and mean BMI of these 26.95 kg/m^2^ (± 4.06) thus characterizing an overweight population.

As regards risk factors for cardiovascular diseases, the following factors were found: predominance of dyslipidemia 46 (73.01%), male sex 44 (69.8%), hypertension 42 (66.66%), followed by sedentary and overweight/obesity 35 (55.55%), hereditary factor for early death 11 (17.46%), diabetes 10 (15.87%) and smoking 3 (4.76%), characterizing the heterogeneity of the sample (Table 1).

**Table 1:** Clinical Data, Type of Treatment and Risk Factors of 63 Patients With Acute Coronary Syndrome, Submitted to Euroqol-5D, on Discharge From Hospital (Salvador, Brazil, 2013)

ACS	n (%)
Entities	
Acute myocardial infarction NSTEMI	25 (39.7)
Acute myocardial infarction STEMI	15 (23.8)
Unstable angina	22 (34.9)
Treatment	
Medical	37 (64.90)
Angioplasty	20 (35.08)
Risk factors	
Dyslipidemia	46 (73.01)
Male sex	44 (69.80)
Hypertension	42 (66.66)
Sedentarism	35 (55.55)
Overweight/obesity	35 (55.55)
Hereditary factors for early death	11 (17.46)
Diabetes	10 (15.87)
Smoking	3 (4.76)

In the studied sample, the Euroqol-5D questionnaire score presented a variation from 63% to 100%, and mean frequency of 89%. Table 2 presents the distribution of the Euroqol-5D domains, in which it was verified that the study population revealed a greater frequency in level 1 (without alterations) in all the domains, personal care 62 (98.41%), followed by mobility 60 (95.23%), habitual activities 57 (90.47%), pain/feeling ill 50 (79.36%) and anxiety/depression 39 (61.90%). As regards level 2 (moderate problems), there was an inversion in the frequency of the domains, with anxiety/depression having greater predominance 24 (38.09%), followed by pain/feeling ill 13 (20.63%), habitual activities 6 (9.56%), mobility 3 (4.76%), with a lower proportion for personal care 1 (1.58%), since this domain portrays activities that are not performed in the hospital environment (occupational and leisure). It is worth pointing out that not one level 3 was mentioned (extreme problem) as regards the domains investigated at the time of discharge from hospital (Table 2).

**Table 2:** Distribution of the Euroqol-5D Domains of the 63 Patients With Acute Coronary Syndrome (Salvador, Brazil, 2013)

Domains	Categories	n (%)
Personal care	Without problems	62 (98.41)
	Moderate problems	1 (1.58)
	Extreme problems	0 (0)
Mobility	Without problems	60 (95.23)
	Moderate problems	3 (4.76)
	Extreme problems	0 (0)
Habitual activities	Without problems	57 (90.47)
	Moderate problems	6 (9.50)
	Extreme problems	0 (0)
Pain/feeling ill	Without problems	50 (79.36)
	Moderate problems	13 (20.63)
	Extreme problems	0 (0)
Anxiety/depression	Without problems	39 (61.90)
	Moderate problems	24 (38.09)
	Extreme problems	0 (0)

**Figure 1:**
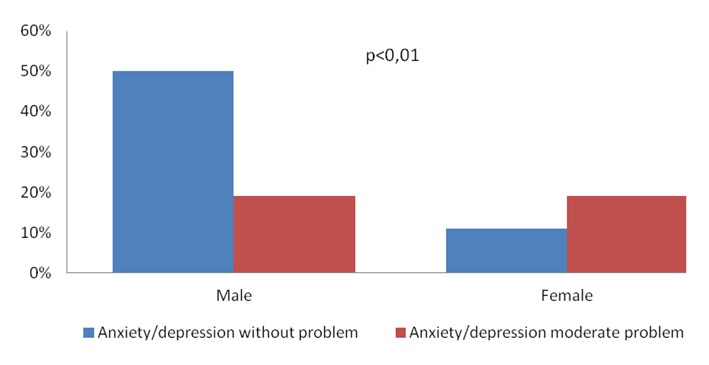
Association between the domain anxiety/depression and sex of the 63 patients with acute coronary syndrome, submitted to Euroqol-5D, Salvador, Brazil, 2013.

When the domains were associated with age, clinical diagnosis, type of treatment and with the risk factors, no statistical significance was verified. The domain anxiety/depression presented statistical significance with associated with sex (P < 0.01) and hereditary factors (P = 0.01) (Fig. 1).

**Figure 2:**
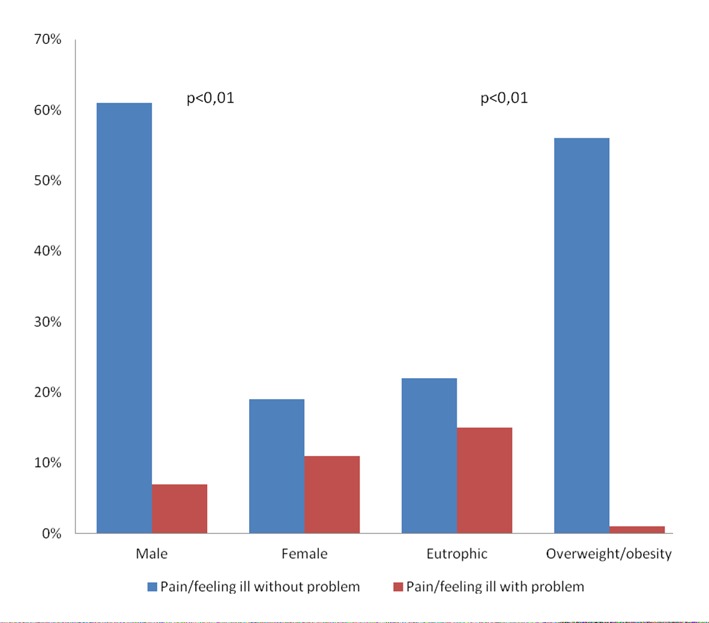
Association between the domain pain/feeling ill and BMI of the 63 patients with acute coronary syndrome, submitted to Euroqol-5D, Salvador, Brazil, 2013.

When the domain pain/feeling ill was verified, a relationship with sex was observed (P = 0.01), and this domain was also associated with the patients’ BMI (P < 0.01) (Fig. 2).

**Figure 3:**
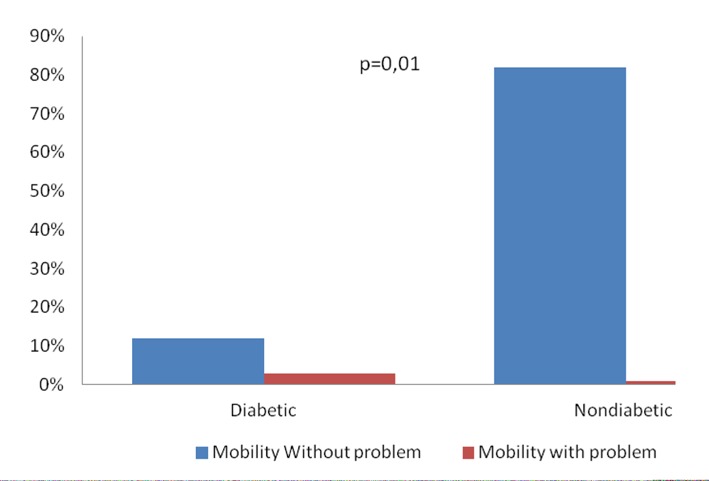
Association between the mobility and diabetes of the 63 patients with acute coronary syndrome, submitted to Euroqol-5D, Salvador, Brazil, 2013.

Of the patients evaluated, 10 presented diabetes, and there was statistical significance with the domain mobility (P = 0.01) (Fig. 3).

## Discussion

Studies have investigated the QoL of patients with ACS; however, these researches evaluated the time from the first month after the acute coronary event and there is a lack of studies that evaluate QoL at the time of discharge from hospital. The present study revealed that the domain anxiety and depression is the one presenting the highest level of compromise, making it difficult for this population to cope during the extra hospital stage, which could compromise the other domains in the long term, particularly that of mobility.

In this study, the patients presented a mean age (59.16 years) similar to that of other researches. As this study concerned a population considered economically active, there is concern about QoL. There was predominance of the male gender, thus confirming the greater risk existent for this population [8, 11]. There was higher prevalence of the diagnosis of AMI NSTEMI, similar to that of an another study that found this diagnosis in 50% of the patients [3]. The patients revealed a greater extent of compromise in the anxiety/depression domain, irrespective of the clinical characteristic and association with risk factors.

In this sample, the men presented higher Euroqol-5D scores than the women, thus characterizing less compromised QoL of the patients of the male sex, being similar to other studies in which the QoL of women was compromised to a greater extent [15, 16]. Moderate problems were related in the anxiety/depression and pain/feeling ill domains, thus corroborating another study in which a greater predominance of anxiety was verified in patients hospitalized due the coronary events [10]. With regard to sex, a study conducted in a hospital environment, with a sample of 345 patients, with both AMI and UA, mean age of 59.45 years, verified a higher prevalence of depression in women with unstable myocardial ischemic syndromes [11], thus differing from the present study, as in this study there was no statistical difference between the sexes, and this difference is believed to have occurred due to the sample size being significantly smaller.

AMI presents the patient with a threat of death, and thus, the individual should become adapted to the new reality, thus generating a high degree of anxiety in these patients [17]. The literature confirms that depression may be a predictor for coronary events, thus being present before the patient is admitted to hospital [10, 18]. Therefore, if the patient presents a previous history of depression, his/her chance of presenting this dysfunction increases. Another hypothesis to consider is the patients’ possible insecurity about the limits on occupational, sexual and leisure activities.

This study verified a relationships of hereditary factors for early death with anxiety/depression; however, no studies with this association were found in the literature, in spite of hereditary factors being an imminent risk for early death. The authors of the present study believe that the fact that the patient had lost a direct family member increases the reluctance about the occurrence of his/her own death.

In a study with a population with a mean age of 44 years that presented chronic pain, it was verified that women related more intense pains, in addition to having musculoskeletal pain more frequently, when compared with men [19]. In the present study women related moderate pain/feeling ill more frequently, when compared with the men. In any case, the moderate feeling of being ill was preponderant over the feeling of musculoskeletal pain, common in the critical population after an acute coronary event.

Diabetic patients at an advanced stage of the disease may present some changes in mobility, which may generate serious consequences, such as falling for example. This pathology may also cause neural and cardiovascular alterations, diabetic neuropathy, diminished visual and auditory acuity, dizziness, polypharmacy, hypoglycemia, among others [20]. In the sample researched, no significant changes in mobility were verified in the diabetic patients. Possibly, the individuals evaluated had not yet presented the functional repercussions expected in the advanced stage of the disease.

The findings in the literature have proved that patients with chronic lumbar pain, with a mean age of 44 years, and with overweight/obesity have a greater tendency to present pains, as there is an overload on the osteo-muscle-articular structure, thus altering the biomechanical equilibrium of the body [19]. The BMI trend of the studied sample is close to the bottom limit of overweight/obesity without involvement in the mobility, pain/feeling ill domains.

The limitation of this study is that it was applied in a single private health center with patients with ACS submitted to RCV1 with progressive exercises, in which the results may not reflect the reality of the population that uses the public health services. Moreover, the Euroqol-5D investigates the domain habitual activities; however, as these patients were restricted to the hospital, they did not perform these activities. In addition, depression was not verified at the time the patients were admitted to hospital.

It was concluded that in this sample, on discharge from hospital after ACS, the patients revealed compromised QoL in the pain/feeling ill and anxiety/depression domains, particularly when associated with risk factors. The need for multidisciplinary care during the extra hospital stage is emphasized.
